# An *in vitro* biofilm model system maintaining a highly reproducible species and metabolic diversity approaching that of the human oral microbiome

**DOI:** 10.1186/2049-2618-1-25

**Published:** 2013-10-02

**Authors:** Anna Edlund, Youngik Yang, Adam P Hall, Lihong Guo, Renate Lux, Xuesong He, Karen E Nelson, Kenneth H Nealson, Shibu Yooseph, Wenyuan Shi, Jeffrey S McLean

**Affiliations:** 1 Microbial and Environmental Genomics, J. Craig Venter Institute, 10355 Science Center Drive, CA 921 21 San Diego, USA; 2 UCLA School of Dentistry, 10833 Le Conte Avenue, CHS Box 951668, Los Angeles, CA 90095, USA; 3 Department of Human Genomic Medicine, J. Craig Venter Institute, 9704 Medical Center Drive, Rockville, MD 20850, USA; 4 Department of Earth Sciences, USC, ZHS 117, Los Angeles, CA 90089, USA

**Keywords:** *In vitro* model, Biofilm, Oral microbiome, Saliva, *Streptococcus*, *Lactobacillus*, Uncultivated bacteria

## Abstract

**Background:**

Our knowledge of microbial diversity in the human oral cavity has vastly expanded during the last two decades of research. However, much of what is known about the behavior of oral species to date derives from pure culture approaches and the studies combining several cultivated species, which likely does not fully reflect their function in complex microbial communities. It has been shown in studies with a limited number of cultivated species that early oral biofilm development occurs in a successional manner and that continuous low pH can lead to an enrichment of aciduric species. Observations that *in vitro* grown plaque biofilm microcosms can maintain similar pH profiles in response to carbohydrate addition as plaque *in vivo* suggests a complex microbial community can be established in the laboratory. In light of this, our primary goal was to develop a robust *in vitro* biofilm-model system from a pooled saliva inoculum in order to study the stability, reproducibility, and development of the oral microbiome, and its dynamic response to environmental changes from the community to the molecular level.

**Results:**

Comparative metagenomic analyses confirmed a high similarity of metabolic potential in biofilms to recently available oral metagenomes from healthy subjects as part of the Human Microbiome Project. A time-series metagenomic analysis of the taxonomic community composition in biofilms revealed that the proportions of major species at 3 hours of growth are maintained during 48 hours of biofilm development. By employing deep pyrosequencing of the 16S rRNA gene to investigate this biofilm model with regards to bacterial taxonomic diversity, we show a high reproducibility of the taxonomic carriage and proportions between: 1) individual biofilm samples; 2) biofilm batches grown at different dates; 3) DNA extraction techniques and 4) research laboratories.

**Conclusions:**

Our study demonstrates that we now have the capability to grow stable oral microbial *in vitro* biofilms containing more than one hundred operational taxonomic units (OTU) which represent 60-80% of the original inoculum OTU richness. Previously uncultivated Human Oral Taxa (HOT) were identified in the biofilms and contributed to approximately one-third of the totally captured 16S rRNA gene diversity. To our knowledge, this represents the highest oral bacterial diversity reported for an *in vitro* model system so far. This robust model will help investigate currently uncultivated species and the known virulence properties for many oral pathogens not solely restricted to pure culture systems, but within multi-species biofilms.

## Background

The human oral cavity harbors a highly diverse and unique microbiome, which exists in a continuously changing environment where pH, organic carbon and oxygen levels fluctuate on a hundred-fold or even a thousand-fold scale within minutes [1,2]. From the first discovery that dental plaque pH will decrease after sucrose consumption and then return to baseline values [3,4], the oral microbial community has been a highly studied system not only because of its health-related significance but also for demonstrating interactions between species and functional analysis of multi-species communities in general [5-7]. Difficulties in studying this complex and structurally heterogeneous environment are multifaceted, and include problems related to the species variability of human subjects, continuous access to samples over time, small sample sizes and complicated ethical issues. This has led to the development of both synthetic consortia biofilm model systems and *in vitro* microcosm plaque from the human natural oral flora by using growth systems, including chemostats [8], the constant-depth film fermentor (CDFF) [9], saliva-conditioned flow cells [10,11] and artificial mouths [12]. Results from these studies show that the multispecies biofilms, containing a hand full of bacterial species, are functionally reproducible with heterogeneous structures and pH behaviors consistent with those of natural plaque [13,14]. As investigators started to understand that the microbial diversity within the oral microbiome is highly important in both health and disease they began to explore multiple species interactions by using mixed-species models consisting of up to 10 defined species. This led to the synthesis of the 'ecological plaque hypothesis,’ which proposes that selection of cariogenic bacteria is directly coupled to alterations in the environment that shifts the balance of the community [15]. According to this hypothesis, if the pH remains below the critical pH (value of 5.5) for demineralization for extended time periods after a carbohydrate pulse, a shift in the bacterial populations to more cariogenic organisms that are acid-producing (acidogenic) and acid-tolerant (aciduric) occurs [16,17]. Another important aspect of this hypothesis is that any species with relevant traits can contribute to the disease process [15,18]. This was also supported by multiple findings that bacterial species, other than well-known pathogens (for example, *Streptococcus mutans*) are present in caries-active sites [19,20]. Also, a recent study shows that one can detect several low-pH active species present in a healthy plaque, which may be responsible for the onset of caries disease [21].

In order to fill in the knowledge gaps in species diversity for these complex communities, the Human Microbiome Project (HMP) [22,23], the Human Oral Microbiome Database (HOMD) [24] and the CORE database [25] have been established. The major goal of the HMP was to characterize bacterial communities that are associated with several different body sites as well as generate a catalog of reference genomes from species derived from human hosts [26]. The HOMD specifically contains information on the prokaryotic species present in the human oral cavity and has the capacity to link sequence data with phenotypic, phylogenetic, clinical and bibliographic information. The curated version of HOMD, published by Dewhirst and colleagues in 2010 contains approximately 619 validated taxa with 1,178 total taxa identified, of which 24% are named, 8% are cultivated but unnamed and 68% represented uncultivated phylotypes [27]. Human oral taxa are defined as sharing 98.5% similarity in 16S rRNA (henceforth abbreviated as 16S) gene sequences [27]. A major hurdle to understanding the oral microbiome is the unknown contribution of the very large uncultivated fraction of the existing bacterial diversity. In fact, the greatest number of the 'most wanted taxa’ (that is, those that have been seen by 16S sequencing but remain uncultivated) targeted for whole genome sequencing in the human body reside in the oral cavity [28,29]. The identity of these oral phylotypes can only be linked to possible functions by using techniques such as nucleic acid-based stable isotope probing (SIP) [21] or single-cell genomics based sequencing approaches [30,31]. Moreover, due to the high taxonomic variability of the oral microbiome between study subjects it is extremely difficult to track species and strains temporally and spatially [23]. Also, small sample sizes that are dictated by availability of volunteers and costs, limit the statistical power needed to detect small, but important differences among communities. Hence, to gain a deeper ecological understanding of the processes that are involved in the gradual succession of healthy oral microbiomes to disease-associated microbiomes, it is important to continue the development of oral microbial model systems where experiments can be conducted in a controlled environment. The advantages with such systems are many as they provide novel opportunities to study microbial community ecology with systems biology perspectives by using global *omics* experimental tools (metagenomics, metatranscriptomics, metabolomics). A model system also allows for generating biological replicates and contributes to the analyses of large samples that are needed to obtain reliable spatial and temporal dynamics data of bacterial populations within a community.

In this study, our aim was to develop a mixed-community biofilm model system comprising the highest possible cultivable bacterial diversity representative of the resident saliva-derived microbiome responsible for plaque formation in the human oral cavity. We used a recently developed growth medium (SHI medium) that previously was shown to support growth of a highly diverse microbiome and that also had a high coverage of species found in the original inoculum saliva samples obtained from healthy adults [32]*.* To evaluate the reproducibility of this *in vitro* model system and to address its overall metabolic potential we applied both conventional community fingerprinting, denaturing gradient gel electrophoresis (DGGE), and next-generation sequencing. The broader taxonomic reproducibility of biofilms growing in two different research laboratories was tested by comparing 16S gene profiles from DGGE. To obtain a broader understanding of the whole biofilm community diversity, deep sequencing of 16S genes via 454-pyrosequencing and whole genome shotgun (WGS) sequencing on the Illumina HiSeq platform was performed. To our knowledge, the model we have developed represents the highest oral bacterial diversity that has been reported for an *in vitro* system thus far. This model can be used to gain a deeper understanding of molecular mechanisms that underlie the evolution of healthy oral microbiomes into disease-associated microbiomes where cariogenic groups such as mutans streptococci and *Lactobaccilli* become more abundant [7,19,33,34]. This model will also aid in the understanding of oral microbial communities by facilitating discovery and functional characterization of known, as well as uncultivated bacteria, within a mixed-species system that is approaching the diversity of *in vivo* conditions. Importantly, it will allow systematic investigation of species, specific genes/domains, gene products and metabolic pathways that define the synergistic and competitive contributions to health and disease in the complex oral microbiome.

## Methods

### Saliva collection

Saliva samples were collected from six healthy subjects, age 25 to 35 years as described by Tian and colleagues [32]. Consents from study subjects, including consent to participate in the study and consent to publish findings from saliva samples were obtained. Subjects were asked to refrain from any food or drink 2 hrs before donating saliva and to spit directly into the saliva collection tube; 5 ml saliva was collected from each person. Ethical approval of all protocols related to saliva collection and experimental research was confirmed by the Institutional Review Board (IRB) (University of California Los Angeles IRB #11-002483). Saliva samples were pooled together and centrifuged at 2,600 g for 10 minutes to spin down large debris and eukaryotic cells. The supernatant was referred to as pooled saliva and used throughout this study. Cell-free saliva was also used for coating wells prior to growing the biofilms as described below.

### Culturing and growth of saliva-derived biofilms using SHI medium

To assess the diversity and the reproducibility of the saliva inoculum pool and our highly diverse *in vitro* grown biofilms we used several incubation approaches and molecular techniques. An overview of the applied conditions is presented in Table 1. Prior to saliva inoculation into SHI medium, (fully described in Tian *et al*. [32]) within a sterile 24-well plate, 200 μl of saliva supernatant was added to each well. This pre-coating of wells allows attached pellicle growth. The plates were then incubated at 37°C with the lid open for 1 hr to dry the saliva coating. Plates were then sterilized under UV light for 1 hr before 10 μl of pooled saliva was inoculated into a pre-coated well containing 10 μl of sucrose (0.5%), 990 μl SHI medium. Plates were incubated at 37°C under anaerobic conditions for 16 hrs to allow biofilm formation. The biofilms were carefully washed twice with a buffered chemically defined medium (CDM), which is fully described in [21]. After the washing steps, biofilms were starved (that is, incubated without carbon source) in fresh CDM medium (pH 7) for 2 hrs in 37°C and incubated under anaerobic conditions (Table 1). After starvation the biofilms were harvested for DNA extraction and 16S gene analyses by using 454-pyrosequencing as explained below. For the temporal analyses of bacterial taxa by using a metagenomics approach, biofilm samples were harvested at the following time points when growing in SHI medium as explained above: 3, 6, 9, 12, 16, and 48 hrs (Table 1). Two replicate biofilm sample wells were harvested at 6 hrs of growth. Note that these biofilms were grown for 48 hrs in total and not starved in minimal medium as compared to the samples that were devoted to 454-pyrosequencing.

**Table 1:** ***in vitro *** **biofilm analyses parameters used in this study to test reproducibility**

**Goal**	**Samples and number of replicate wells**	**Incubation conditions prior to DNA extraction**	**Community profile technique**
Address batch and well reproducibility	Batch 1 × 1	Step I: 16 hrs growth in SHI medium	16S-454 pyrosequencing
	Batch 2 × 2		
	Batch 3 × 3		
	Saliva × 3	Step II: 2 hrs starvation in CDM	
		No incubation	
Address reproducibility between research laboratories	With sucrose × 2 without sucrose × 2	16 hrs growth in SHI medium with or without sucrose	DGGE
Address temporal taxonomic diversity during biofilm development	Time course (hrs) = 3,6,9,12,16,48	48 hrs growth in SHI medium	WGS metagenomes Illumina HiSeq

CDM, chemically defined medium; DGGE, denaturing gradient gel electrophoresis; WGS, whole genome shotgun.

### Glucose and pH monitoring of biofilm growth medium

After the 2-hr period of starvation, 1 ml of 0.5% glucose in fresh CDM (pH 7) was added to each biofilm well. Glucose levels were then measured in the biofilm growth medium by using the glucose-specific TRUE2go blood monitoring system (CVS Pharmacy, Inc., Woonsocket, RI, USA). A 3-μl sample was withdrawn from a glucose-designated sample well throughout the biofilm incubation. This sample was added to a sterile plastic surface inside the anaerobic chamber prior to applying the test strip, which was then mounted onto the electronic glucose-meter reader. Three replicate samples were analyzed at incubation time points: zero, 2, 4, 5, 6 hrs. The lower limit for glucose detection of the TRUE2go blood monitoring device is 20 mg/dl, which was reached after 5 hrs of incubation. Biofilm-growth medium pH was monitored in near real-time within replicate pH-designated incubation wells and measured by combining pH Laboratory Electrodes (EW-05990-65, Cole-Parmer, Court Vernon Hills, IL, USA) with a wireless sensor network platform consisting of a pH transmitter (UWPH-2-NEMA, OMEGA, Stamford, CT, USA) and a pH receiver (UWTC-REC1, OMEGA). pH was monitored in the same fashion in the biofilm growth wells that were incubated for 48 hrs in SHI medium and subjected to metagenomics analyses. pH measurements were monitored and downloaded onto a computer using TC central software for UWTC (OMEGA). Real-time pH was recorded for 48 hrs every 30 seconds within each growth well.

### DNA extraction and processing of pyrosequencing data

DNA was isolated as described in [21] using the DNeasy Blood and Tissue Kit (Qiagen Inc., Maryland, USA) and eluted in a final volume of 200 μl water. Biofilms representing the batch-2 samples were a part of a stable isotope probing (SIP) time-series study (unpublished) in which a series of samples were subjected to ^13^C-labelled glucose amendments in the CDM buffer as described in McLean *et al*. 2012 [21]. However, the batch-2 samples described in this study were not fed labeled ^13^C-glucose, as they served as controls and were collected immediately (time point zero hours of incubation). The DNA from the SIP-treated biofilm replicate samples (batch-2 samples) was separated by centrifugation against a cesium chloride (CsCl) density-gradient [21]. The sample processing of these particular samples was conducted as follows: (1) entire DNA was extracted from each sample and loaded into the gradient solution; (2) gradient formation was achieved by centrifugation at 265,000 × g for 66 hrs in a Beckman VTi65 rotor (Beckman Coulter, Inc., Fullerton, CA, USA); (3) fractions were collected (400 μl) and the DNA was isolated using a YM-100 Microcon column (Millipore, Billerica, MA, USA); (4) columns were washed four times with TE buffer and purified DNA was eluted into 50-μl volumes. Biofilm samples representing batch 1 and batch 3 were not subjected to ultracentrifugation. After genomic DNA extraction and quantification, all samples were prepared for 16S amplification and titanium-based 454-pyrosequencing at the J. Craig Venter Institute (JCVI) Joint Technology Center (JTC). Genomic DNA sample concentrations were normalized to 2 to 6 ng/μl. The V3 to V5 region of 16S genes was amplified according to the previously developed protocol available at http://www.nature.com/nature/journal/v486/n7402/extref/nature11209-s1.pdf[23]. Using each sample's individual barcodes, the 454 sequence data were deconvolved into the respective samples. After trimming the bar codes, low-quality and short sequences (<100 bp) were removed by using the JCVI 16S pipeline. Subsequently, the remaining filtered reads were aligned against the SILVA database of 16S to verify that the reads were indeed 16S. The Chimera Slayer tool was used to filter out potentially chimeric reads [32].

### PCR-DGGE analysis

Reproducibility of 16S gene diversity was tested by two different research laboratories, JCVI, San Diego, CA, USA and the School of Dentistry, University California Los Angeles (UCLA), CA, USA. Both research laboratories carried out saliva inoculation from the same saliva pool into SHI medium prepared independently at each laboratory. Samples were incubated and washed at the different locations as described above. Genomic DNA extractions, PCR amplification and DGGE analyses were carried out at the UCLA laboratory. Amplification of bacterial 16S genes and DGGE analysis was carried out as described in a previously published protocol [32]. The universal primer set Bac1 and Bac2 [35] was used to amplify an approximately 300-bp internal 16S fragment of the 16S gene.

### Biofilm and saliva operational taxonomic unit (OTU) diversity in pyrosequencing libraries

To compare OTU diversity between saliva and biofilm samples, the following analysis steps were conducted using the MOTHUR software [36]: (1) quality filtered fragment reads were assigned to OTUs in 97% sequence identity level; (2) distances of samples in datasets were calculated in terms of OTU profile; (3) Yue and Clayton metrics was applied, which measures structure dissimilarities between communities [37]; (4) the obtained Yue and Clayton matrix was used to calculate and visualize sample similarities using correspondence analysis (CA) [38] in the ADE-4 software [39].

### Identification and phylogenetic analysis of 16S genes

To identify previously defined oral bacterial taxa in the biofilm and saliva samples, a reference alignment was initially created by downloading 16S RefSeq Extended Version 1.1 from Human Oral Microbiome Database (HOMD) [24], which consists of 1,647 sequences that includes named, unnamed and uncultivated phylotypes. By using this reference sequence set we generated 1,642 non-redundant sequences by using cd-hit-est [40] with 100% sequence identity and 95% alignment coverage for the shorter sequence. The 1,642 sequences were aligned by cmalign in the Infernal package [41]. A phylogenetic tree was generated with RAxML [42] using this alignment. By using blastn [43], our sequence reads from the saliva and biofilm samples were aligned to the HOMD reference sequences. Matches at 97% sequence identity cutoff and 95% sequence coverage were considered. Match counts for saliva and biofilm samples were scaled in base-10 log. Reference tree and the counts were visualized by using the online interactive tree of life (iTol) software [44]. In the figure, color ranges were shown at phylum level. Bar charts next to leaf labels show log scale match counts for saliva and *in vitro* biofilm samples, respectively (Figure 2). A bubble chart was generated based on the base-10 log transformed match counts to show sample similarities for human oral taxon (HOT) designations (named, unnamed and uncultivated phylotypes) in biofilm samples batch 2, well 1 to well 2, and batch 3, well 1 to well 3.

### Metagenomic analyses of biofilms

WGS sequencing was performed on total DNA that was extracted from biofilms growing in individual growth wells at 3, 6, 9, 12, 16, and 48 hrs of growth in SHI medium. Replicate libraries were prepared from two biofilms representing 6 hrs of growth. WGS sequences (fragment and paired-end reads) were obtained from the Illumina HiSeq platform, quality trimmed and filtered using CLC workbench software v. 6.0.1 (CLCbio, Aahus, Denmark). The following CLC-parameters were applied during paired read sequence trimming and quality control: quality score setting: NCBI/Sanger or Illumina Pipeline 1.8 and later, minimum distance: 180, maximum distance: 250. The trimmed reads were subjected to sequence assembly by using the CLC workbench (CLCbio). ORF calling and annotations were performed on the contigs obtained from the CLC workbench according to the JCVI prokaryotic metagenomics pipeline [45]. A summary of metagenomic read numbers, contig numbers and identified ORFs for each sample is presented in Additional file 1: Table S1. Metagenome annotations were uploaded for comparative analyses with metagenomes from the HMP on METAREP [46]. HMP assembly annotations, available via the HMP through the JCVI-supported METAREP (http://www.jcvi.org/hmp-metarep) representing supragingival plaque, keratinized gingiva, mid vagina, posterior fornix and anterior nares body sites were used for hierarchical cluster analysis together with biofilm samples representing 12 and 16 hrs of growth. Cluster analyses were performed using the Multiple Experiment Viewer (MeV) (version. 4.8.1) (http://www.tm4.org/mev.html) the average linkage setting and Pearson correlation as distance measure. Bacterial community compositions were profiled for all time points using the metagenomic read-based analysis tool MetaPhlAn (version 1.7.7) [23,47]. This computational tool relies on clade-specific marker genes from 3,000 reference genomes and performs estimations of organism relative abundance in terms of numbers of cells rather than fraction of reads.

## Results and discussion

### Meeting the challenges with oral *in vitro* model systems

The goal of this study was to develop and validate with deep sequencing, a novel robust *in vitro* model system representative of the naturally complex oral microbiome. In previous studies of biodiverse model biofilm systems a major approach has been to construct synthetic plaque-like consortia represented by a handful of plaque species [18,48-50]. Other methods represent inoculating and growing bacteria from natural saliva in plaque microcosms such as the artificial mouth [12,51-54]. The latter studies of natural saliva focus solely on physiological responses and do not consider taxonomic diversity at 16S gene level. Studies that derive from less diverse synthetic plaque communities show intriguing examples of how certain oral bacteria form advantageous partnerships that are necessary for growth of other species and that these partnerships sometimes are also observed *in vivo*[55-57]. Each model system has strengths, limitations and difficulties. However, they all represent better study models than the uncontrollable environment of the mouth. A major challenge when using any oral model system is to maintain a representative diversity of the indigenous oral microbiome. This was specifically addressed in a previous study by Tian and colleagues [32], who developed a growth medium (SHI medium) that supported a remarkably high number of oral taxa *in vitro* from a small number of human saliva samples which were inoculated and grown as biofilms. Here we employed this medium with the goal to select for the highest possible diversity representative of the oral cavity, high reproducibility and stability at the species level in this *in vitro* biofilm model.

### Pooling saliva samples from different sampling subjects prior to inoculation

To meet the above stated goals it was necessary to pool saliva samples from several humans to acquire enough biomass for the startup of multiple *in vitro* sample growth wells in parallel. This enabled us to study numerous replicate samples simultaneously by using multiple analyses approaches such as 454-pyrosequencing of 16S genes, measurement of pH and sugar levels during growth and future comparative studies on the saliva pool, such as metabolomics and metatranscriptomics. Another important aspect of the pooling of saliva samples was that it allowed us to domesticate previously uncultivable community members, such as those belonging to the candidate phylum TM7 and the genus *Porphyromonas*[58]. Several of these community members would most likely have been missed if we had studied saliva from one single person due to the high taxonomic variability between humans [33]. However, a limitation of using a pool of saliva samples deriving from several individuals as inoculum in the *in vitro* model system is that the native community integrity is lost as the samples are mixed together. Most likely, each person who participated in this study harbored a unique saliva microbiome, which may not be accurately reflected from a community diversity perspective here. Clearly, in future studies it is important to address how a single person’s microbiome evolves taxonomically and metabolically over time in our *in vitro* model and to what extent species within a native community accomplish certain functions only in their native setting as compared to when they are mixed with other community members from other individuals.

### Moderating the sugar concentration for the highest diversity

Initially, through our deep sequencing experiments we sought to confirm the most appropriate concentration of additional sucrose needed in the SHI medium to best represent the saliva-derived oral community that our previous DGGE results showed more qualitatively [32]. The responses to two carbon source concentrations added to the SHI medium (0.1% and 0.5% sucrose) and a control with no sucrose were tested by sequencing 16S genes from the resulting biofilms grown *in vitro*. We found that 0.5% sucrose was able to maintain the saliva-derived diversity in biofilms most similar to the saliva inoculum in terms of the genera observed (see Additional file 2: Figure S1). In contrast, the no-sucrose biofilm was less diverse and was dominated by a single *Streptococcus* species, *S. mitis*. The 0.1% sucrose-amended sample was similar to the one grown in the presence of 0.5% sucrose but had a higher proportion of subgingival community members, such as *Porphyromonas*. This confirmed that 0.5% sucrose was the better choice for our model and that the taxonomic diversity responds rapidly to environmental changes.

### Physiological responses to carbohydrate pulse are similar to *in vivo* plaque

After establishing the growth conditions, we sought to confirm that the general physiological response of a plaque biofilm is maintained *in vitro* (that is, similar to the *Stephan curve*) [3,4]. This particular response has been acknowledged for decades as the classic Stephan curve where oral acidogenic bacteria rapidly metabolize fermentable carbohydrates producing acidic byproducts and then the pH returns to baseline [3,4]. When we initiated biofilm incubation with 0.5% glucose in fresh CDM the pH started to drop instantly (see Additional file 3: Figure S2). pH was neutral in the beginning of the incubation and decreased to 4.5 after 4 hours. At 6 hours it had reached its lowest level (4.2) and then it started to recover. Glucose concentrations followed the same falling pattern and after 5 hours were below the detection limit (20 mg/dl) showing a rapid turnover of this substrate by bacteria in the biofilms (see Additional file 3: Figure S2). Rapid glucose utilization and conversion to lactate and acetate was also shown in this CDM for *S. mutans* and fresh plaque samples derived from children using nuclear magnetic resonance techniques [21,59]. These results confirm that our *in vitro* model system shows a similar physiological response to a glucose challenge as previously described for dental plaque and *in vitro*-tested salivary sediments [60,61]. Overall, the response of this model biofilm is consistent with dental plaque containing species capable of lowering and also raising the pH.

### Reproducibility between research laboratories

After optimizing the community towards a higher diversity, 16S gene amplification and DGGE analyses were performed from biofilm samples that shared the same pool of saliva but were grown by two different research laboratories (UCLA School of Dentistry, and JCVI) to test technical reproducibility (see Additional file 4: Figure S3). Although the saliva inoculum was shared, completely independent media reagents and growth procedures were carried out in the respective labs. The resulting replicate gel images were aligned and 16S band patterns were compared for samples that had been incubated with or without 0.5% sucrose, showing that despite the variations between labs, the resulting community composition was highly reproducible. The majority of the DGGE bands showed similar fluorescence intensity across replicates and only one major band was missing in the replicate samples deriving from the JCVI laboratory (see Additional file 4: Figure S3).

### Reproducibility across batches (alpha diversity) and between wells within a batch (beta diversity)

**Figure 1:**
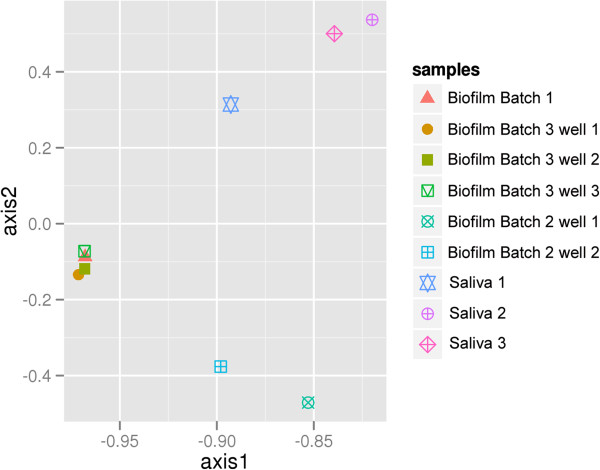
**Correspondence analysis showing reproducibility and 16S profile similarities within biofilm and saliva samples.** Axis 1 explains 47% of the variation in the dataset; axis 2 explains 19% of the variation. Replicate biofilm samples representing batches 1 and 3 cluster closely together whereas batch-2 biofilms that derive from an SIP experiment cluster more distantly along the first ordination axis. Saliva-derived replicates (1 to 3) also show similar 16S diversity.

To evaluate the reproducibility between batches and between growth wells within a batch for our model system in-depth, we analyzed 454-pyrosequencing data obtained from biofilms cultivated on different dates (batches 1, 2, and 3) and grown in replicate growth wells from the same freshly thawed saliva pool (Table 1). Included were two biofilm samples (batch 2, well 1 and 2) that had been subjected to 66 hours of DNA centrifugation, which served as a control in a parallel SIP experiment (unpublished). The biofilm samples batches 1, 2, and 3 were all grown and processed on different days to assess the reproducibility of batches at different time points; replicate growth wells from batches 2 and 3 were compared to address the variation between biofilm growth wells. Similarities in OTU diversity between replicates from all biofilm batches and from saliva inoculum samples were estimated using CA (Figure 1). In CA, the saliva samples were similar and clustered together but separately from the biofilm samples. The biofilm libraries that derived from batch 2 clustered separately from biofilm samples representing batches 1 and 3 that formed a separate cluster. The difference between batch 2 and the other biofilm samples was most likely due to the fact that the batch 2 samples were centrifuged over an extensive period of time in a CsCl gradient, which according to many SIP studies will separate high GC DNA from low GC and thus likely resulted in a skewed 16S profile. Regardless of this difference however, these centrifuged samples were highly similar to each other, and the community profiles for replicate biofilm samples were highly reproducible, which supports their potential to serve as replicate samples for experiments in which a particular treatment is compared.

### 16S gene diversity in pooled saliva and biofilm samples

**Figure 2:**
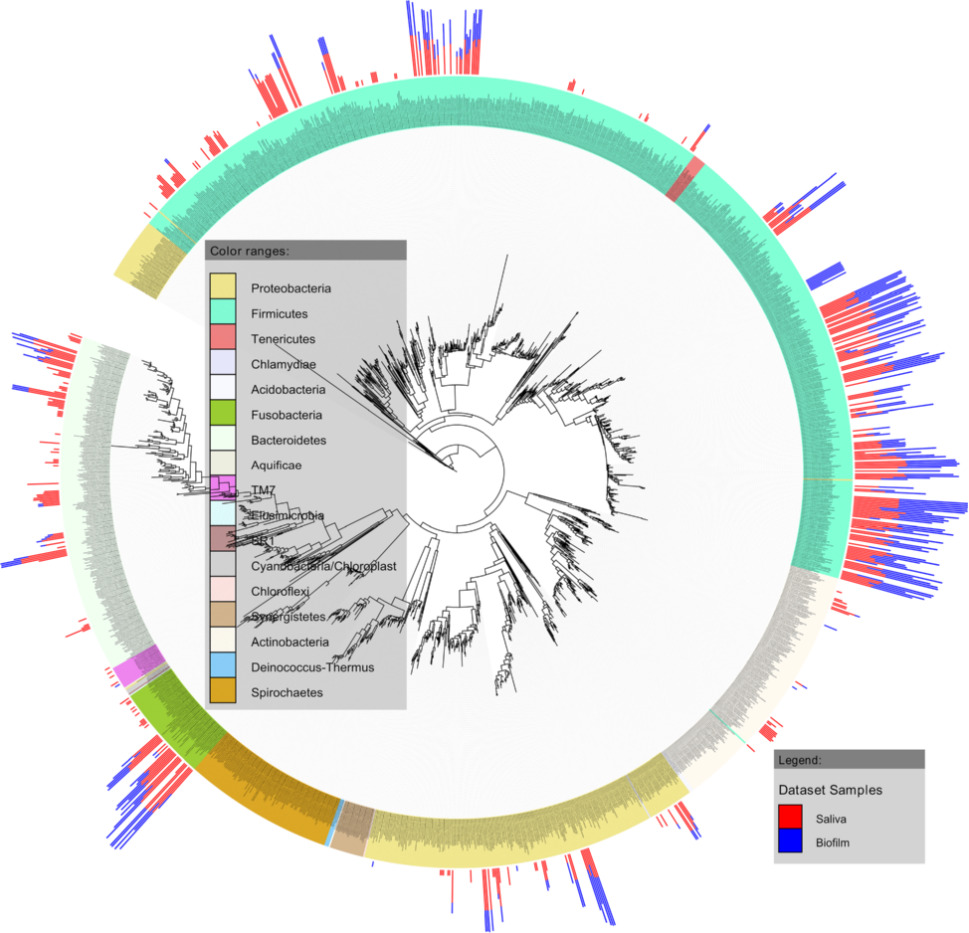
**Phylogenetic tree based on 1,642 Human Oral Microbiome Database (HOMD) reference sequences.** 16S genes that were obtained from pyrosequencing in this study were matched based on sequence homology with HOMD reference sequences. Red and blue bars indicate normalized relative abundance counts of human oral taxon (HOT) designations that were identified in the saliva and biofilm samples, respectively. Sequence matches between reference sequences and query sequences were counted at 97% sequence identity cutoff and 95% sequence coverage.

**Figure 3:**
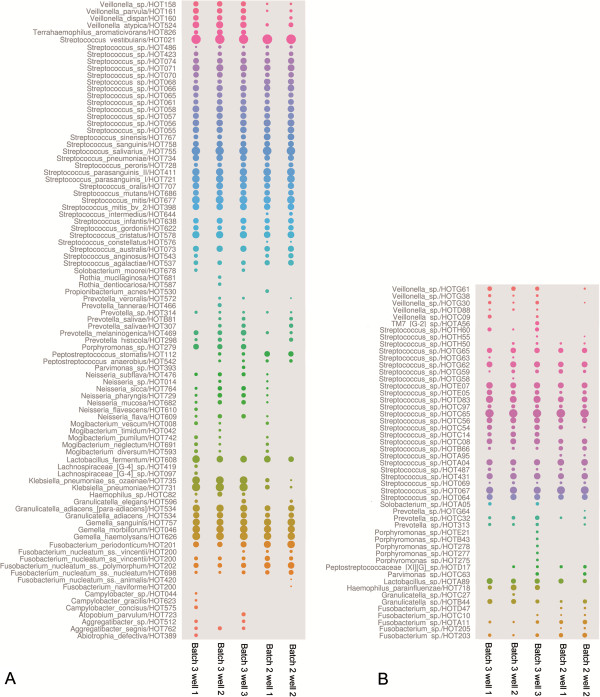
**Bubble charts showing 16S profile reproducibility at human oral taxon (HOT) designation level (98.5% sequence similarity) in replicate biofilm growth wells.** Batches 2 and 3 were grown at different time points and batch 2 was also subjected to an additional centrifugation step during DNA extraction. Cultivated species, both unnamed and named batches, according to HOMD classification are presented in **A**. Uncultured phylotypes are presented in **B**. Read-abundances were log transformed and all identified HOTs are presented here. Bubble sizes correspond to relative abundance values calculated from log-transformed 16S read-abundance data for each HOT. Standard deviation in HOT abundance between wells from the same batch ranged between 0.1% and 3% whereas standard deviation was higher (0.3 to 8%) between batches.

In order to assess the bacterial phylogenetic diversity in saliva and biofilm samples at a higher resolution, reference sequences from the HOMD were used to taxonomically classify 16S genes from this study (Figure 2; Additional file 5: Table S2). Matching HOT designations and their log-transformed abundance values were aligned on the outside of an in-house constructed HOMD-phylogenetic tree (see Methods) at their corresponding branch tips. Within the HOMD-classified 16S genes that were obtained from this study more strain diversity (that is, sequences showing >98.5% sequence similarity) was observed but is not included here. In total, 6 phyla and 41 genera of bacteria were identified in the different samples (singletons included) (Figure 2). These corresponded to 264 HOT designations of which 131 also could be detected in one or several biofilm samples. Several of the dominant genera (for example, *Streptococcus*, *Veillonella, Prevotella*) were equally abundant in samples grown at different time points and in samples grown in different incubation wells, showing that the biofilms grown *in vitro* are highly reproducible (Figure 3). This was also shown by the low standard deviations of HOT abundance values between replicate libraries, which ranged between 0.1% and 8% across replicate samples. Of particular note, 51 uncultivated HOT designations belonging to the *Streptococcus*, *Granulicatella*, *Haemophilus*, *Lactobacillus*, *Parvimonas*, *Peptostreptococcus*, *Prevotella*, *Solobacterium*, *Fusobacterium*, *Veillonella, Porphyromonas* and TM7 G-2 genera could be identified in the biofilm growth wells (Figure 3B). Identification of the most abundant genera in saliva and biofilm samples was done by removing genera that encompassed HOT-designations that contributed with two hundred or fewer 16S genes counts, corresponding to <0.3% of total 16S gene abundance (Table 2). Two genera (*Streptococcus* and *Veillonella*) were detected in biofilms when using the latter criteria while nine were identified when including the rare community members (those that were represented with ≤200 16S gene counts). These results show that most of the diversity in the *in vitro* biofilms could be attributed to the *Streptococcus* and *Veillonella* genera, which is in line with previous findings of healthy oral microbiomes [33].

**Table 2:** Numbers of human oral taxa (HOT) within the most dominant genera in saliva and biofilm samples

**Bacterial phyla (bold) and genera (italics)**	**Number of HOT identified**	**Number of HOT identified *in vitro***	**Number uncultivated**		**Number uncultivated**	
			**Phylotypes (P)**		**Phylotypes (P)**	
	**Saliva**^**a**^	**Biofilm**^**a**^	**HOMD**^**a**^		**HOMD**^**b**^	
			**Saliva**	**Biofilm**	**Saliva**	**Biofilm**
**Firmicutes**						
*Streptococcus*	25 (58)	38 (58)	6	11	25	25
*Veillonella*	3 (9)	2 (9)	1	1	5	5
*Parvimonas*	4 (6)	0 (2)	1	ND	1	1
*Mogibacterium*	4 (5)	0 (5)	ND	ND	ND	ND
*Gemella*	3 (3)	3 (3)	ND	ND	ND	ND
*Peptostreptococcus*	3 (3)	0 (3)	3	ND	ND	ND
*Granulicatella*	1 (2)	1(2)	ND	ND	2	2
*Klebsiella*	0 (2)	2 (2)	ND	ND	ND	ND
*Lactobacillus*	0 (0)	1(2)	ND	ND	ND	1
**Bacteroidetes**						
*Prevotella*	3 (38)	0 (11)	1	ND	3	3
*Porphyromonas*	1 (8)	0 (6)	ND	ND	4	4
**Fusobacteria**						
*Fusobacterium*	4 (12)	2 (11)	2	ND	5	5
**Proteobacteria**						
*Neisseria*	5 (11)	0 (7)	ND	ND	ND	ND
*Campylobacter*	1 (5)	0 (3)	1	ND	ND	ND
**TM7:**						
TM7 (G-1)	0 (2)	0 (0)	ND	ND	2	ND
TM7 (G-2)	0 (0)	0 (1)	ND	ND	ND	1
TM7 (G-3)	0 (1)	0 (0)	ND	ND	1	ND

HOT designations were defined according to the Human Oral Microbiome Database (HOMD) classification scheme. (), Total numbers of HOT, including singletons, are presented inside brackets. ^a^Uncultivated phylotypes were identified based on the HOMD classification. The numbers of phylotypes representing HOT designations with >200 16S gene counts (corresponding to >0.3% of total 16S counts) are presented in these columns. ^b^All HOT designations (including those that were represented by 1 to 200 copies of16S gene reads). ND, no uncultivated HOMD representative was detected.

#### Streptococcus

Members belonging to the genus *Streptococcus* are predominant bacterial species of human saliva and supragingival biofilms [23,62,63], which also were observed for the biofilm *in vitro* model system in this study. The most dominant HOT designations belonged to *S. vestibularis, S. salivarus*, *S. mitis*, *S. parasanguinis* and a variety of understudied *Streptococcu*s sp. strains (Figure 3). *S. vestibularis* HOT-021 contributed approximately 40% to the total 16S abundance in all replicate biofilm samples, whereas *S. salivarus* (HOT-755) and the uncultivated phylotype *Streptococcus* sp (HOT-C65) both contributed 10% each. *S. vestibualris* is a normal inhabitant of the oral cavity and has rarely been associated with human disease except in a few cases of infectious endocarditis, early neonatal sepsis and bacteremia in cancer patients [64]. Also, *S. vestibularis* was previously shown to produce only low levels of caries in rats when compared to other *Streptococcus* species (for example, *S. salivarus*) [65]. The impacts on oral human health of *S. salivarus* span a broad range from being strongly cariogenic [66,67] to caries-protective by hydrolyzing urea to ammonia [68-70]. *S. parasanguinis* strains HOT-721 and HOT-711 were also abundant in the *in vitro* biofilms but contributed only half of the 16S sequence read-abundance when compared to *S. salivarus* and *Streptococcus* sp. HOT-C65. The ecological role of *S. parasanguinis* in oral health is also poorly understood but due to its capacity to ferment multiple carbohydrates to lactate and other organic acids it is considered to be a moderately acid-tolerant organism and a cariogenic species [71,72]. Previous studies show that it has been significantly associated with both caries in young children and healthy oral flora [73,74]. Our *in vitro* biofilm model supports growth of several *S. parasanguinis* strains in concert and can be used to test these relationships further.

#### Lactobacilli

Members belonging to the *Lactobacillus* genus are found in low abundance in supragingival samples from healthy subjects and are thought to be late colonizers in microbial succession [23]. They are commonly observed in advanced caries and are isolated on low pH agar [75-77]. In the *in vitro* biofilms two *Lactobacillus* species (*L. fermentum* HOT-608 and *Lactobacillus* sp. HOT-A89) could be identified at a low abundance (average 0.8%), which is relatively close to the abundance of *L. fermentum* (0.2%) observed in the clone libraries used to generate the HOMD. This represents an opportunity to challenge the model and track the increase in these cariogenic species, simulating a transition from a healthy community to a more disease-like state.

#### Veillonella atypica/Veillonella dispar/Veillonella parvula/Veillonella sp

The presence of these understudied species in our biofilm model gives us future opportunities to learn more about the role of *Veillonella* in oral community succession and caries. In previous studies of *Veillonella* its ecological role has been unclear and laboratory studies show that effects of pH on its growth can be mixed [78,79]. It has also been suggested that the presence of *Veillonella* could possibly be used as a predictor of future caries in caries-free children and that it has a close and complex relationship to the pathogen *S. mutans,* which also was detected in this study [20,80].

#### Fusobacterium

Bacterial community members belonging to the *Fusobacterium* genus contributed approximately 10% to the total 16S gene diversity in the biofilm communities. Like most of the other oral community members they are known to be associated both with the normal human oral flora and also with certain oral diseases [81]. Their ability to grow in many different habitats can be explained by their broad metabolic versatility as they can obtain energy from fermentation of a broad range of simple sugars and amino acids, free or in the form of peptides [81]. Three strains representing the invasive host pathogen *F. nucleatum* were identified here as well as a strain representative of *F. peridonticum*[82,83].

Several previously identified oral pathogens were observed at notably low 16S gene abundance in all samples (for example, *S. mutans* HOT-686, *Rothia mucilaginosa* HOT-681, *Abiotrophia defectiva* HOT-389, *Atopobium rimae* HOT-750, *Porphyromonas catoniae* HOT-283*, Prevotella melaninogenical* HOT-469) (Figure 2) indicating the pathogenic potential present in samples derived from healthy oral subjects. A representative of the elusive candidate Phylum TM7 [G-2] that was previously identified in both healthy human subjects and in subjects with periodontal disease and other inflammatory mucosal infections [84-86] was also present at a low abundance in a biofilm sample (Figure 3).

### Diversity indices in 454-sequencing libraries

Although classification-based methods have the benefit of being associated with a reference taxonomy, these methods cannot account for the diversity that is missing from the reference databases. Therefore, we applied an OTU-based approach to complement the above classification approach. These results show that OTU richness was lower in biofilm samples compared to saliva samples, ranging from 65 OTUs in the batch-2 well-1 sample to 156 OTUs in the batch-3 and batch-2 samples (Table 3). In line with these results, the Shannon entropy (H) index was also slightly higher in the saliva-derived samples (4.39 to 4.72) compared to the biofilm samples where H varied from 4.11 to 4.23. Although it is clear that the biofilms at this selected time point do not fully capture every species found in a saliva sample, the *in vitro* biofilms in this study contain the highest 16S diversity identified so far within an *in vitro* oral model system.

**Table 3:** Diversity and count estimates of 16S genes in pyrosequencing libraries after sequence trimming

**Sample**	**Number of raw 16S rRNA fragment reads**	**Number of OTUs after pre-cluster analyses**^**b**^	**Shannon entropy**	**Shannon evenness**
Biofilm batch 1	6011	132	3.7436	0.7620
Biofilm batch 2 well 1^a^	5731	107	3.6971	0.7960
Biofilm batch 2 well 2^a^	8538	158	4.1868	0.8200
Biofilm batch 3 well 1	4835	154	4.1622	0.8284
Biofilm batch 3 well 2	5166	146	4.1082	0.8289
Biofilm batch 3 well 3	4778	148	4.2334	0.8683
Saliva 1	6956	208	4.7209	0.8544
Saliva 2	6885	243	4.7209	0.8544
Saliva 3	3799	179	4.5234	0.8828

Biofilm batches 1, 2 and 3 correspond to replicate biofilm samples grown at different time points. The biofilm batch-2 library was generated from stable isotope probing experiments (see Methods). ^**a**^Wells 1, 2 and 3 correspond to replicate technical samples collected from different sample wells. ^b^Numbers of operational taxonomical units (OTUs) after precluster-analyses using the SILVA reference alignment and the MOTHUR software.

### Metabolic reproducibility and temporal taxonomic shifts as shown by WGS

**Figure 4:**
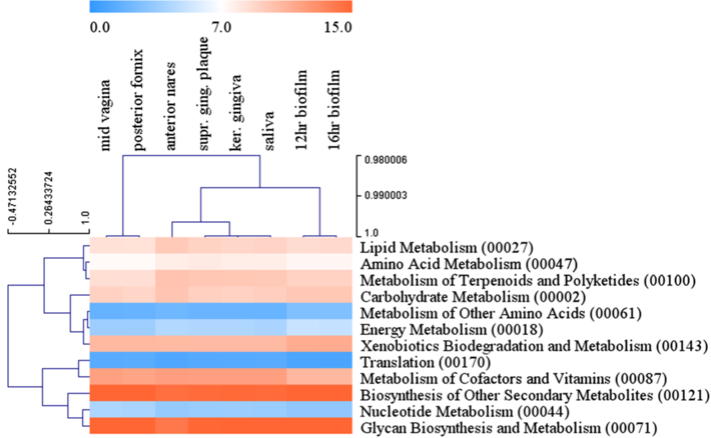
**Metabolic profile comparisons of Human Microbiome Project (HMP) oral metagenomes, other HMP-body sites and the *in vitro *biofilm metagenomes.** The METAREP tool was used to compare Kyoto Encyclopedia of Genes and Genomes (KEGG) super pathways representing the 12- and 16-hrs-old *in vitro* biofilms from this study with HMP-oral metagenomes representing supragingiva, keratinized gingiva and saliva samples from healthy subjects. These oral metagenomes were also compared to HMP metagenomes from other body sites, that is, anterior nares (external position of nostrils), mid vagina and posterior fornix (area behind lower portion of uterus) by using hierarchical cluster analyses of ORF abundance data for each sample. Colored bar in the top indicates relative abundance in percentage of annotated ORFs that fall within each KEGG super pathway (blue, approximately 0%; white, approximately 6%; red, approximately 16%).

Using a WGS approach to further investigate the *in vitro* biofilm community, we confirmed that the type and proportions of metabolic pathways present in our *in vitro* biofilm model, here represented by two annotated metagenomes, were in strong agreement with those recently reported for supragingival plaque, keratinized gingiva (gum tissue closest to the teeth) and saliva samples in the HMP studies of 242 healthy human subjects (Figure 4) [23,33]. This was shown in the Kyoto Encyclopedia of Genes and Genomes (KEGG) pathway and enzyme classification comparison-analyses where major pathways (for example, secondary metabolite biosynthesis, glycan biosynthesis and metabolism, metabolism of cofactors, vitamins, terpenoids, polyketides, lipids, amino acids, energy, nucleotides and translation) were more similar for the oral sites compared to anterior nares (external position of nostrils), mid vagina and posterior fornix (area behind lower portion of uterus) body sites. Comparing the enzyme classification of the body sites at lower levels further supports the overall functional similarities with supragingival plaque (Figure 4; Additional file 6: Figure S4 and Additional file 7: Figure S5). These findings are in line with the HMP study of healthy Western subjects which revealed that there is large variation in carriage of taxa (community structure) but stable metabolic pathways exist across individuals for many body sites including supragingival plaque [23]. Here the 16S datasets and our WGS data show that our *in vitro* biofilm model has a different but stable taxonomic diversity, which harbors a highly similar metabolic potential representative of the human oral cavity. Taken together, both the results of this study and the HMP study suggests that some variation in the relative proportions of taxa in a model system such as this may be tolerated, as the functional capabilities (carbohydrate utilization and pH buffering) of the community may remain similarly stable and allow us to gain some fundamental understanding as to what is happening *in vivo*.

### Biofilm development as studied using metagenomics

**Figure 5:**
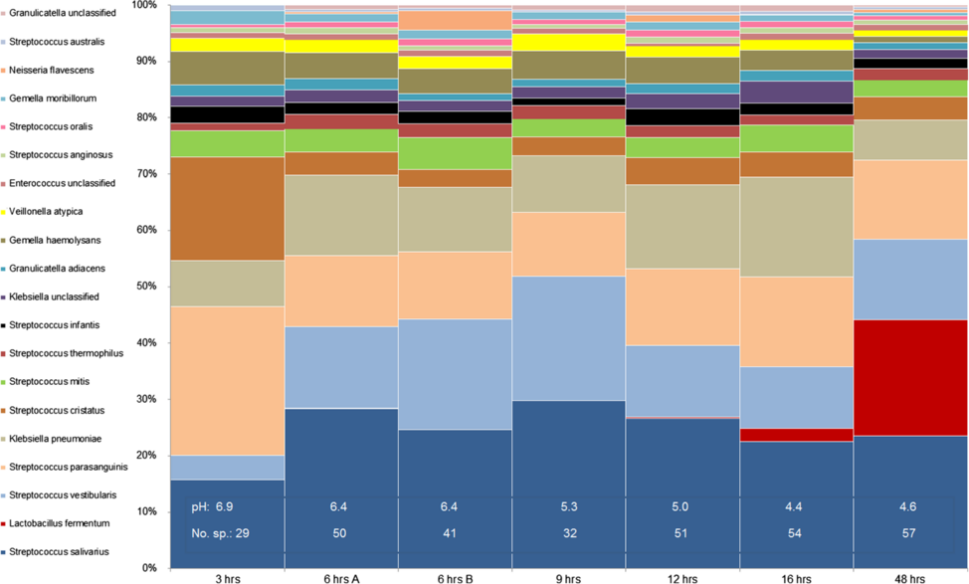
**Temporal metagenomic read-abundance profiles from MetaPhlan analyses of bacterial species.** Relative abundance of paired-end reads that were classified at bacterial species-level based on clade-specific marker genes from 3,000 reference genomes [47]. pH at the different time points is shown in white text inside the bar graph. No pH measurement was available for 48 hrs (N/A). Numbers of estimated bacterial species from MetaPhlan analyses are shown inside the bar graph (No. sp.).

The relative abundance of bacterial species shifted in biofilms over a time period of 48 hours of growth in sucrose-supplemented SHI medium (Figure 5). Initially, at 3 hrs of growth, *S. parasanguinis* represented the most dominant community member, contributing approximately 25% to the total read abundance. *S. cristatus* and *S. salivarus* were also dominant members at this stage, each contributing approximately 15% to the total read abundance. As pH decreased, the abundance of *S. parasanguinis* and *S. cristatus* decreased until reaching 16 hrs. At 48 hrs *Lactobacillus fermentum* had increased significantly in relative abundance whereas *Klebsiella pneumonia* had decreased from circa 20 to 10% between 16 and 48 hrs of biofilm development. pH, which was measured over a time period of 48 hrs, reached its lowest levels of pH 4.4 at 16 hrs when the abundance of the acidogenic and cariogenic species *Lactobacillus fermentum* started to increase (Figure 5). It is likely that inhibition of metabolism, due to low pH and possibly carbohydrate limitation, starts to impact growth of some community members between 12 and 16 hrs and this leads to a shift in the community structure. Moreover, the number of bacterial species that was identified using the MetaPhlan tool was the highest after 48 hrs of growth, showing that this species diversity was still increasing over time (Figure 5). Growth development and bacterial activity are important aspects that need to be addressed in future studies by sampling with higher temporal resolution in the early stages of growth and coupling this to measurements of live biomass and possibly transcription changes.

Taken together with the 16S gene sequencing results showing that *L. fermentum* was a rare species in the early stages of *in vitro* growth and that *L. fermentum* is considered a late colonizer of oral plaque [20] as the pH decreases, it is likely that the *in vitro* biofilm community was at an intermediate state of succession at the time point of 16S gene analysis (16 hrs). With this information, we can possibly modulate the community to resemble an early or later stage of development. Likewise, it would be interesting to test how the biofilm develops and differs if a saliva pool of diseased subjects is used. Its usefulness in human health research could span a wide range, as saliva pools with different origin could be collected and inoculated based on the tested hypotheses (for example, inoculum can represent saliva from diseased human subjects or healthy subjects with highly different microbiomes). Overall, these results reflect that this model system maintains a high number of species that can be used to study both the healthy (pH balancing) and acid-tolerant species associated with disease.

## Conclusions

The principal challenge in most sub-fields of microbiology still relates to the microbial cultivation barrier. In the human oral cavity the proportions of uncultured species are lower (approximately 60%) than in other environments [87-89]; however, the missing species are a significant impediment to the study of human health. The use of an *in vitro* model system with a highly complex bacterial diversity that also supports growth of uncultivated oral species is highly desirable, as it can be manipulated and studied in a controlled environment. To our knowledge, the model we developed here is the first validated system at the species and gene level that fulfills these criteria and that can be readily used to target changes in taxa, regulation of metabolic pathways and signaling molecules, using next-generation sequencing and omics methodologies. For the 131 HOT designations that were identified in the *in vitro* biofilms 48 fully sequenced genomes are available through HOMD, showing that we now have the ability to perform in-depth genome analyses at the community level. Longitudinal studies of an *in vitro* model system such as this will generate deeper fundamental knowledge of the mechanisms regulating bacterial taxonomic diversity and community functions in both health and disease. Specifically, such a model system will help facilitate experimental approaches that seek answers to questions concerning caries-related diseases and how oral pathogens can be eradicated. We hope that it will be used in future research as a tool to understand and combat the development of oral disease.

### Availability of supporting data

The datasets supporting the results of this article are included within the article (and its additional files).

## Abbreviations

16S: 16S rDNA gene that encode 16S ribosomal RNA; 16S RefSeq Extended Version 1.1: A dataset that contains 1,647 16S rRNA gene sequences representing all currently named and unnamed oral taxa as well as taxa that have not yet been assigned with a taxon identity; 454-pyrosequencing: A method of DNA sequencing based on 'sequencing by synthesis’ which was licensed to 454 Life Sciences (Banford, CT). It involves taking a single strand of DNA and then synthesizing its complementary strand enzymatically. The pyrosequencing method is based on finding the activity of the DNA polymerase; Bp: Base pairs; CA: Correspondence analysis is a multivariate statistical technique that applies to categorical data. It provides a means of displaying or summarizing a set of data in two-dimensional graphical form; cd-hit-est: A computer program designed to quickly group nucleotide sequences into clusters that meet a user-defined similarity threshold; CDM: A chemically defined medium that simulates saliva medium for oral bacterial species; DGGE: Denaturing gradient gel electrophoresis is a form of electrophoresis which uses a chemical gradient to denature the samples as it moves across an acrylamide gel. It is applied to separate nucleic acids based on fragment size and nucleotide content; HMP: The National Institute of Health-funded Human Microbiome Project had the goal to characterize microbial communities found at multiple human body sites and to look for correlations between changes in microbial and human health; HOMD: The Human Oral Microbiome Database provides comprehensive information on the approximately 700 prokaryote species that are present in the human oral cavity; HOT: A human oral taxon is defined with a unique ID number in HOMD that is linked to its unique 16S rRNA gene phylotype for which phenotypic phylogenetic, genomic, clinical and bibliographic information is available; iTo: The Interactive Tree of Life software is an online tool for the display and manipulation of phylogenetic trees; JCVI: (Joint Craig Venter Institute); KEGG: Kyoto Encyclopedia of Genes and Genomes; OTU: Operational taxonomic unit species distinction in microbiology. A 97% rDNA sequence similarity threshold for classifying bacteria within the same or different OTUs was used in this study; PCR: Polymerase chain reaction; RAxM: A statistical method for maximum likelihood phylogeny estimation; SHI medium: A previously developed blood-based growth medium that supports growth of a diversity of oral bacteria; SILVA: A database that provides comprehensive quality checked and regularly updated datasets of aligned small and large subunits ribosomal RNA (rRNA) sequences for all three domains of life; SIP: Stable isotope probing is a technique that identifies active microorganisms that assimilate particular carbon substrates and nutrients into cellular biomass. It is an important technology for assigning metabolic functions to microbial communities. Following the incubation of an environmental sample with stable-isotope labeled compounds extracted nucleic acid is subjected to density gradient ultracentrifugation and gradient fractionation to separate nucleic acids of differing densities; UCLA: University of California Los Angeles; WGS: Whole genome shotgun sequencing is a sequencing technology where DNA is broken up randomly into numerous small segments which are sequenced to obtained reads

## Competing interests

Dr. W Shi is a part time Chief Science Officer of C3 Jian Inc., which has licensed technologies from UC Regents that could be indirectly related to this research project. The other authors declare no competing interests.

## Authors’ contributions

AE, RL, XH, KEN, KHN, SY, WS, and JSM designed the experiments, AE, APH, LG, and XH collected and processed the samples, AE, YY, SY, and JSM analyzed the data and AE and JSM drafted the manuscript. All authors helped edit the manuscript. All authors read and approved the final manuscript.

## Supplementary Material

Additional file 1: Table S1 Summary of whole genome shotgun (WGS) data for metagenomics analyses of *in vitro* biofilms at 3, 6, 9, 12, 16 and 48 hrs of growth. Click here for file

Additional file 2: Figure S1 Correspondence analyses of bacterial community structures based on 16S gene analyses; levels of sucrose that served as a carbon substrate during the first 16 hrs of biofilm growth in SHI medium. High sugar and low sugar levels correspond to 0.5 and 0.1% sucrose, respectively. The inoculum sample corresponds to the pooled saliva sample. 16S rRNA community profiles in the high-sugar biofilms were more similar to the natural saliva samples. Click here for file

Additional file 3: Figure S2 Glucose and pH responses in biofilm growth wells during growth in minimal chemically defined medium (CDM). (**A**) Glucose concentration in replicate biofilm samples after spiking samples with 0.5% glucose at time point zero hrs. After glucose concentrations were below 20 mg/dl (6 hrs) they could no longer be detected (dashed line) and were considered as 0 mg/dl. (**B**) Replicate pH profiles of biofilm samples after glucose spiking. pH levels decreased during the first six hours of incubation in parallel with glucose consumption. pH recovery could be observed after 6 hrs of glucose spiking. Click here for file

Additional file 4: Figure S3 Polymerase chain reaction and denaturing gradient gel electrophoresis (DGGE) from two different research laboratories. DGGE gel images showing reproducibility of bacterial 16S gene profiles representing two replicate DNA extractions (left and right panels) from the saliva-derived inoculum cultured in SHI medium with (w/ sucrose) and without sucrose (w/o sucrose) at two different research laboratories (JCVI and UCLA). DGGE band patterns were similar within and between research laboratories and only one DGGE band was missing in replicate samples as indicated by arrows. Click here for file

Additional file 5: Table S2 16S rRNA read counts in saliva and *in vitro* biofilm samples and their corresponding genome match in the Human Oral Microbiome Database (HOMD). Click here for file

Additional file 6: Figure S4 Hierarchical cluster analyses of Kyoto Encyclopedia of Genes and Genomes (KEGG) pathways representing 12- and 16-hrs-old *in vitro* biofilms in this study and Human Microbiome Project (HMP) metagenomes corresponding to post fornix, anterior nares (Nares), buccal mucosa, subgingiva, keratinized gingiva, saliva, supragingiva and keratinized gingiva samples from healthy subjects. Note that saliva metagenomes were derived from the HMP and not from this study. Colored bar in the top indicates relative abundance in percentage of annotated ORFs that fall within each KEGG pathway (blue, approximately 0%; white, approximately 10%; red approximately 20%). The 20 most abundant KEGG pathway hits are presented here. A total of 147 hits were identified in all metagenomes using METAREP. Click here for file

Additional file 7: Figure S5 Hierarchical cluster analyses of METAREP enzyme classifications (Level 4). Metagenomes representing 12- and 16-hrs-old *in vitro* biofilms in this study and Human Microbiome Project (HMP) metagenomes corresponding to post fornix, anterior nares (Nares), buccal mucosa, subgingiva, keratinized gingiva, saliva, supragingiva and keratinized gingiva samples from healthy subjects were included here. Note that saliva metagenomes were derived from the HMP and not from this study. Colored bar in the top indicates relative abundance in percentage of annotated ORFs that fall within each Kyoto Encyclopedia of Genes and Genomes (KEGG) pathway (blue, approximately 0%; white, approximately 0.01%; red, approximately 0.5%). The 29 most abundant enzyme hits are presented here. A total of 1591 enzymes were identified in all metagenomes at METAREP enzyme level 4. Click here for file
