# Supplementary figures and images for: An in vitro biofilm model system maintaining a highly reproducible species and metabolic diversity approaching that of the human oral microbiome
Source: Microbiome. 2013 Oct 2;1:25. doi: 10.1186/2049-2618-1-25 (PMC3971625; doi:10.1186/2049-2618-1-25)

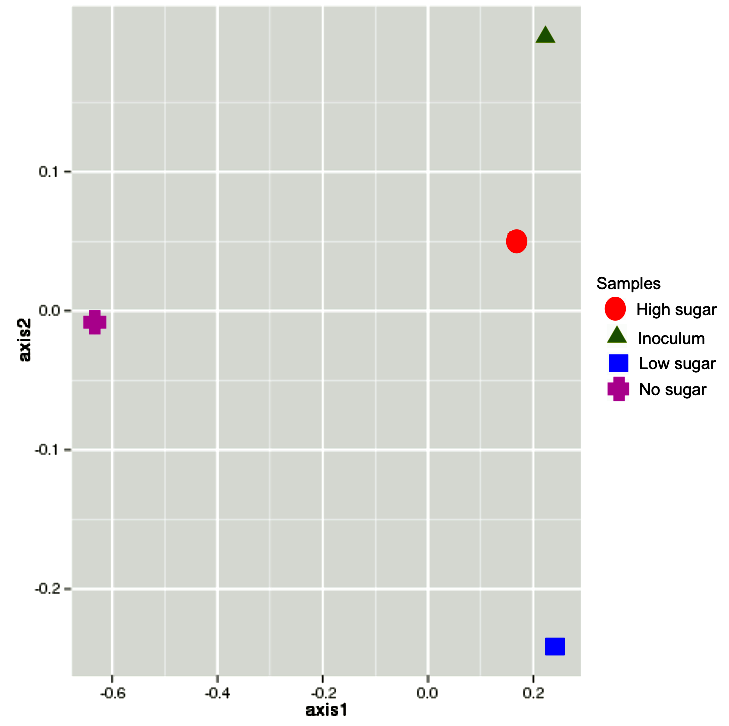

Supplement: Additional file 2: Figure S1 — Correspondence analyses of bacterial community structures based on 16S gene analyses; levels of sucrose that served as a carbon substrate during the first 16 hrs of biofilm growth in SHI medium. High sugar and low sugar levels correspond to 0.5 and 0.1% sucrose, respectively. The inoculum sample corresponds to the pooled saliva sample. 16S rRNA community profiles in the high-sugar biofilms were more similar to the natural saliva samples. [file 2049-2618-1-25-S2.tiff]

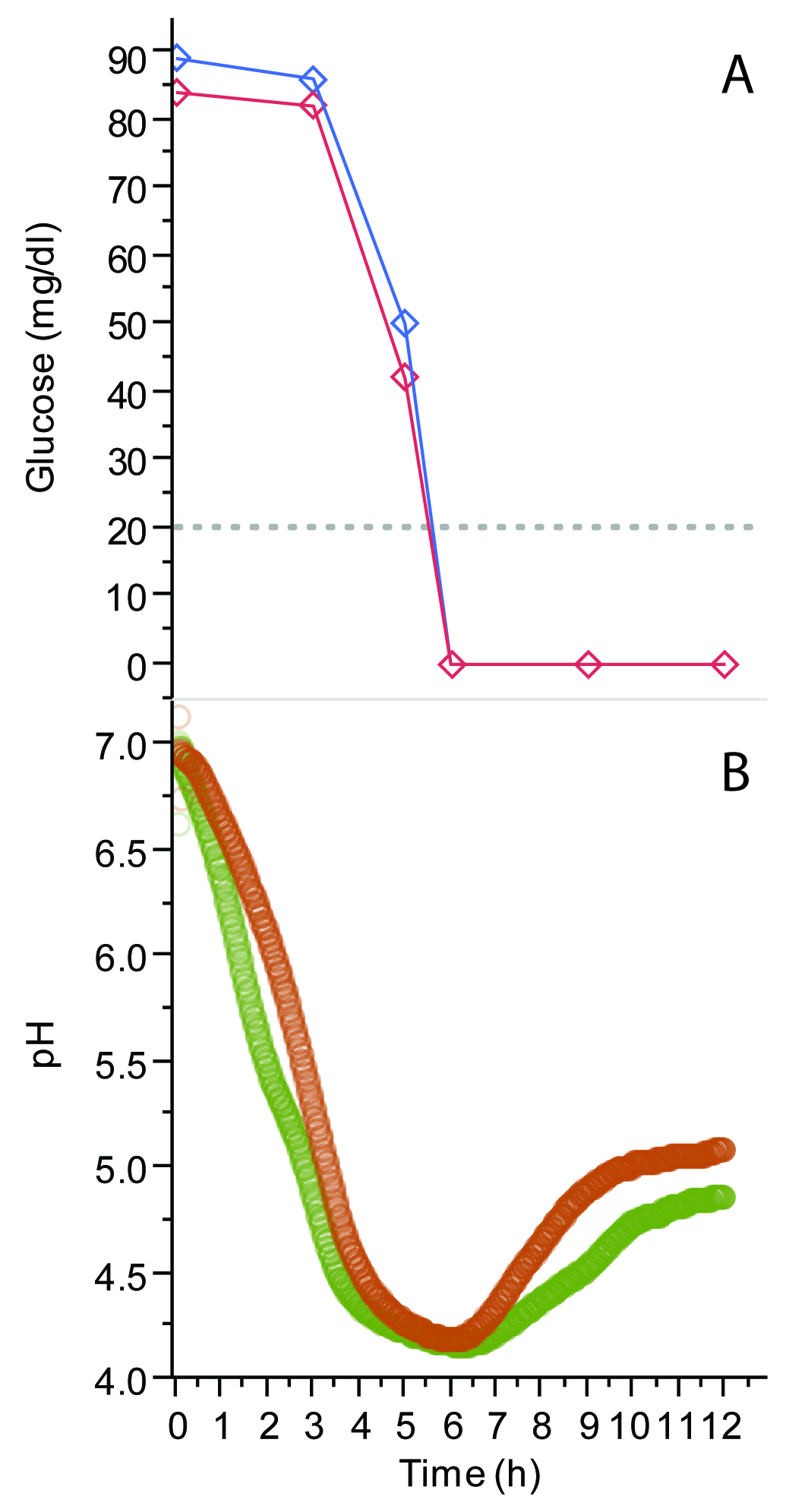

Supplement: Additional file 3: Figure S2 — Glucose and pH responses in biofilm growth wells during growth in minimal chemically defined medium (CDM). (A) Glucose concentration in replicate biofilm samples after spiking samples with 0.5% glucose at time point zero hrs. After glucose concentrations were below 20 mg/dl (6 hrs) they could no longer be detected (dashed line) and were considered as 0 mg/dl. (B) Replicate pH profiles of biofilm samples after glucose spiking. pH levels decreased during the first six hours of incubation in parallel with glucose consumption. pH recovery could be observed after 6 hrs of glucose spiking. [file 2049-2618-1-25-S3.tiff]

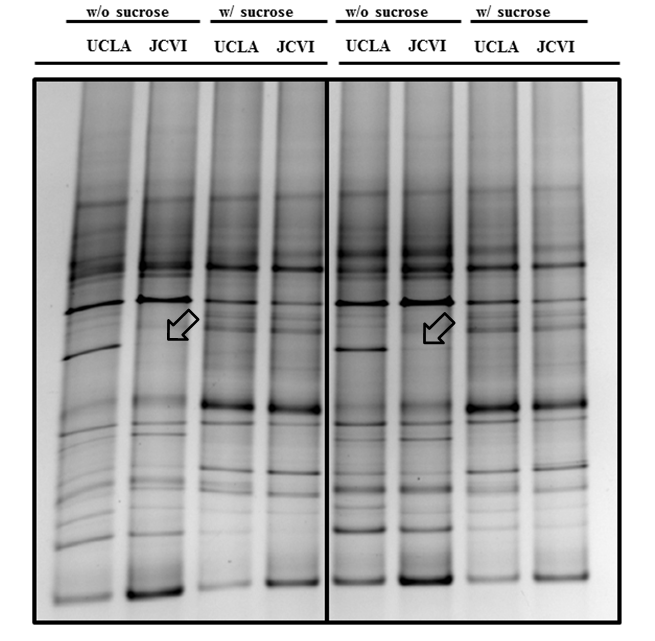

Supplement: Additional file 4: Figure S3 — Polymerase chain reaction and denaturing gradient gel electrophoresis (DGGE) from two different research laboratories. DGGE gel images showing reproducibility of bacterial 16S gene profiles representing two replicate DNA extractions (left and right panels) from the saliva-derived inoculum cultured in SHI medium with (w/ sucrose) and without sucrose (w/o sucrose) at two different research laboratories (JCVI and UCLA). DGGE band patterns were similar within and between research laboratories and only one DGGE band was missing in replicate samples as indicated by arrows. [file 2049-2618-1-25-S4.png]

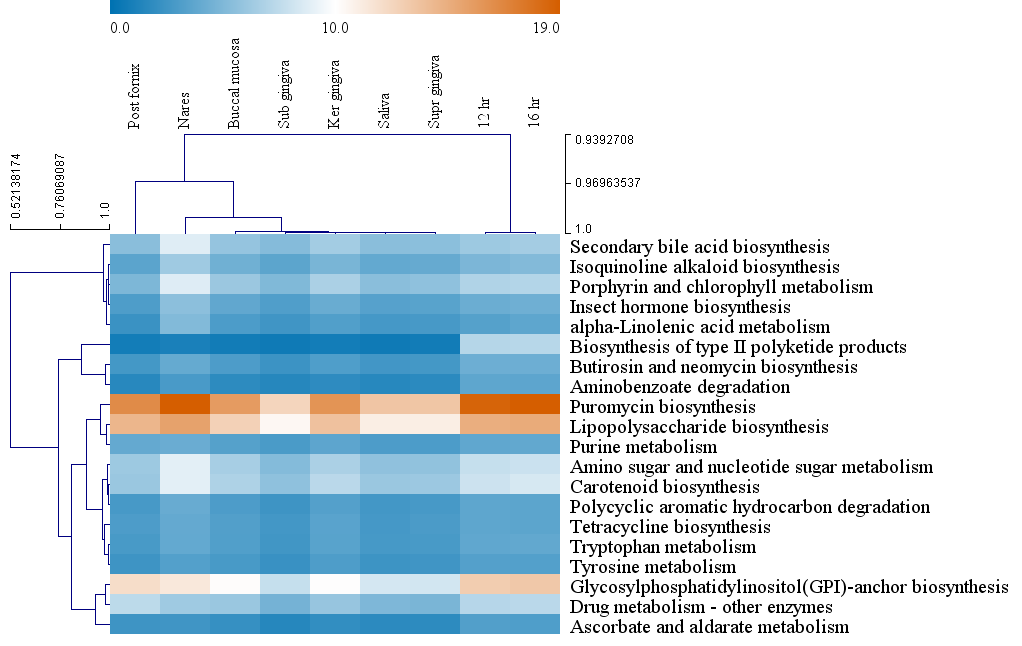

Supplement: Additional file 6: Figure S4 — Hierarchical cluster analyses of Kyoto Encyclopedia of Genes and Genomes (KEGG) pathways representing 12- and 16-hrs-old in vitro biofilms in this study and Human Microbiome Project (HMP) metagenomes corresponding to post fornix, anterior nares (Nares), buccal mucosa, subgingiva, keratinized gingiva, saliva, supragingiva and keratinized gingiva samples from healthy subjects. Note that saliva metagenomes were derived from the HMP and not from this study. Colored bar in the top indicates relative abundance in percentage of annotated ORFs that fall within each KEGG pathway (blue, approximately 0%; white, approximately 10%; red approximately 20%). The 20 most abundant KEGG pathway hits are presented here. A total of 147 hits were identified in all metagenomes using METAREP. [file 2049-2618-1-25-S6.tiff]

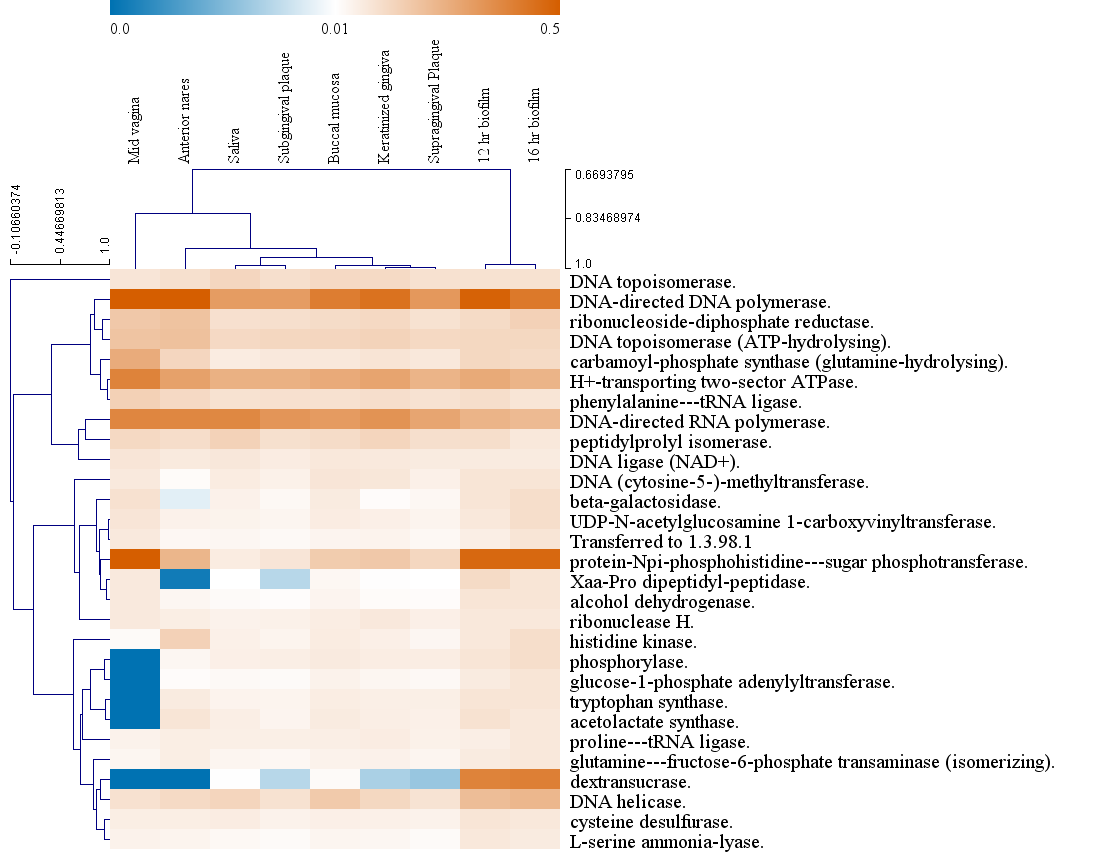

Supplement: Additional file 7: Figure S5 — Hierarchical cluster analyses of METAREP enzyme classifications (Level 4). Metagenomes representing 12- and 16-hrs-old in vitro biofilms in this study and Human Microbiome Project (HMP) metagenomes corresponding to post fornix, anterior nares (Nares), buccal mucosa, subgingiva, keratinized gingiva, saliva, supragingiva and keratinized gingiva samples from healthy subjects were included here. Note that saliva metagenomes were derived from the HMP and not from this study. Colored bar in the top indicates relative abundance in percentage of annotated ORFs that fall within each Kyoto Encyclopedia of Genes and Genomes (KEGG) pathway (blue, approximately 0%; white, approximately 0.01%; red, approximately 0.5%). The 29 most abundant enzyme hits are presented here. A total of 1591 enzymes were identified in all metagenomes at METAREP enzyme level 4. [file 2049-2618-1-25-S7.tiff]
